# Spatial and seasonal variation in the prevalence of *Anaplasma phagocytophilum* and *Borrelia burgdorferi* sensu lato in questing *Ixodes ricinus* ticks in Norway

**DOI:** 10.1186/1756-3305-6-187

**Published:** 2013-06-20

**Authors:** Atle Mysterud, William Ryan Easterday, Lars Qviller, Hildegunn Viljugrein, Bjørnar Ytrehus

**Affiliations:** 1 Centre for Ecological and Evolutionary Synthesis (CEES), Department of Biosciences, University of Oslo, P.O. Box1066, Oslo, NO-0316, Norway; 2 Norwegian Veterinary Institute, P.O. Box 750 Sentrum, Oslo, NO-0106, Norway

**Keywords:** Anaplasma, Borrelia, Dilution effects, Host competence hypothesis, Ixodes ricinus, Lyme Borreliosis Spirochaetes, Prevalence, Red deer, Rodents, Ticks

## Abstract

**Background:**

Understanding the variation in prevalence of *Borrelia burgdorferi* sensu lato (Lyme Borreliosis Spirochaetes, LBS) and *Anaplasma phagocytophilum* (causing tick-borne fever in ruminants and human granulocytic ehrlichiosis) in ticks is vital from both a human and an animal disease perspective to target the most effective mitigation measures. From the host competence hypothesis, we predicted that prevalence of LBS would decrease with red deer density, while prevalence of *A*. *phagocytophilum* would increase.

**Methods:**

Based on a sample of 112 adult and 686 nymphal *Ixodes ricinus* ticks collected with flagging during questing from 31 transects (4–500 m long) corresponding to individual seasonal home ranges of 41 red deer along the west coast of Norway, we tested whether there were spatial and seasonal variations in prevalence with a special emphasis on the population density of the most common large host in this area, the red deer (*Cervus elaphus*). We used a multiplex real-time PCR assay for detection of *A*. *phagocytophilum* and LBS.

**Results:**

Prevalence of LBS was higher in adult female ticks (21.6%) compared to adult male ticks (11.5%) and nymphs (10.9%), while prevalence was similar among stages for prevalence of *A*. *phagocytophilum* (8.8%). Only partly consistent with predictions, we found a lower prevalence of LBS in areas of high red deer density, while there was no relationship between red deer density and prevalence of *A*. *phagocytophilum* in ticks. Prevalence of both bacteria was much higher in ticks questing in May compared to August.

**Conclusions:**

Our study provides support to the notion that spatial variation in host composition forms a role for prevalence of LBS in ticks also in a northern European ecosystem, while no such association was found for *A*. *phagocytophilum*. Further studies are needed to fully understand the similar seasonal pattern of prevalence of the two pathogens.

## Background

There is general agreement that *Ixodes ricinus* ticks have expanded in Europe in the last decades, probably due to climate change and potentially other factors such as increased host density and landscape changes [1]. Among emerging infectious diseases linked to climate change in Europe, the tick-borne disease Lyme borreliosis, caused by certain genotypes of the *Borrelia burgdorferi* sensu lato complex, hereafter called Lyme Borreliosis Spirochaetes (LBS), is one of the ones having the highest potential of severity to human society [2]. Another important disease agent in ticks is *Anaplasma phagocytophilum*. Some variants of this bacterium cause tick-borne fever (TBF) in domestic ruminants [3,4], regarded by many as the most widespread tick-borne infection in animals in Europe [5]. Some genetic variants of *A*.*phagocytophilum* also cause granulocytic anaplasmosis in humans [6], horses [7] and companion animals [3,8].

Risk of exposure to vector-borne pathogens is, among other factors, influenced by the abundance of the vector and the prevalence of the pathogen within the vector population [9]. Knowledge about variation in both tick abundance and pathogen prevalence is, therefore, necessary to understand disease risk [10]. It is a common statement that the increase in tick-borne disease in humans is associated with an overall increase in deer populations and closer contact between humans and deer [11]. This conclusion is based on the role of deer as important hosts for adult ticks, providing enough blood to ensure production of a large number of eggs in the adult female tick, resulting in high abundance of ticks. Deer species, therefore, may play a major role in tick abundances both in Europe [12,13] and North America [14,15], though not in all cases [9,16]. However, deer do not seem to be competent transmission hosts for LBS [9,17-20]. Actually, the innate immune system of cervids may even kill LBS in infected ticks feeding on them [21,22]. This is supported by the lower prevalence of LBS reported from ticks collected on roe deer (*Capreolus capreolus*) and moose (*Alces alces*) compared to ticks collected in the landscape [23]. A study comparing islands in Norway also documented a lower prevalence in areas of high deer density [24]. However, several studies have found either no relationship or a positive relationship between roe deer density and prevalence in continental European ecosystems [25-27]. Rather, passerine birds are the main reservoir for *B*. *garinii*[28] and rodents for *B*. *afzelii*[29]. These are the two most common genospecies of LBS circulating in ticks in Europe, followed by *B*. *burgdorferi* sensu stricto and *B*. *valaisiana*[30], while prevalence of *B*. *burgdorferi* sensu stricto is lower in Norway [31]. Both *B*. *afzelii* and *B*. *garinii*, which are the dominating genotypes found in questing ticks in Norway [30], can cause human disease and hence LBS as a group are important and are our focus here.

The prevalence of both LBS [32-34] and *A*. *phagocytophilum*[35] varies geographically, but prevalence may not follow the same pattern as tick abundance, as host species may differ largely in both abundance and in their degree of reservoir competence for the actual strains of the bacteria (LBS: [36]; *A*. *phagocytophilum*: [4,37]). As such, it has been proposed that deer, although they are important hosts for the ticks, reduce the prevalence of LBS in ticks. However, some researchers argue that this only happens at unrealistic high densities of deer [38]. The potential dilution role of deer hosts may be counteracted by their role in vector augmentation, increasing the number of larvae feeding on reservoir-competent small mammals and some passerine birds. Knowledge about the extent to which deer density is responsible for “dilution effects” is thus one aspect necessary to understand variation in Lyme-disease risk [39]. The role of host composition for prevalence of LBS is well established in general [39,40], but studies from northern Europe are scarce. However, direct or indirect co-feeding transmission may enable deer to transmit LBS in spite of being incompetent hosts [41-44]. If important, one would expect co-feeding transmission to be more common in areas of high abundance of ticks, due to the increased likelihood of uninfected ticks feeding temperospatially close to infected ticks. Higher prevalence in areas with higher abundance of ticks has been previously reported in Sweden [45].

*A*. *phagocytophilum* was previously regarded as a bacterium circulating between a wide range of hosts. However, recent research suggests multiple, more or less distinct subpopulations with co-existing, but more or less independent transmission cycles restricted to one or a few host species [4,46]. The role of European cervids in the transmission cycles of *A*. *phagocytophilum* are not completely understood, but high prevalence of infection [4,47], presence of similar genotypes, both in deer and questing ticks in the same location [46] and establishment of persistent, subclinical infection after experimental inoculation [48] suggest that deer may function as competent reservoir hosts for some of the subpopulations. Consequently, a high population density of red deer (*Cervus elaphus*) should be expected to be associated with high prevalence of *A*. *phagocytophilum* in questing ticks. In accordance with this, higher prevalence of *A*. *phagocytophilum* was found in islands of higher deer density in Norway [24]. Co-feeding transmission has, to our knowledge not been regarded as playing a role in the transmission cycles of *A*. *phagocytophilum*. However, infected and non-infected ticks feeding on the same animal at the same time should increase transmission rate, at least in rodents, where the level of bacteraemia is regarded to be low and life span short [3]. High infestation intensity and frequency also seem to increase the bacteraemia in ruminants [49] and may, as such, possibly facilitate transmission in these species.

In this study, we have analyzed the variation in prevalence of *B*. *burgdorferi* sensu lato (LBS) and *A*. *phagocytophilum* using PCR in 112 questing adult and 686 nymphal ticks sampled from 31 transects corresponding to individual red deer home ranges along the west coast of Norway, with high densities of red deer in some areas. Our study area comprised of a wide range of environmental gradients (coast-inland, low-high elevation, flat-steep, low-high density of red deer; low-high density of ticks), we assessed how all of these gradients affected the prevalence of both pathogens, which has not been previously carried out. We specifically tested the following hypotheses: *The host competence hypothesis* (or dilution hypothesis) predicts reduced prevalence of LBS in areas of high red deer density, but a positive link to prevalence of *A*. *phagocytophilum*. *The tick abundance hypothesis* predicts increased prevalence of both LBS and *A*. *phagocytophilum* in areas with high abundance of ticks. This can result from higher transmission efficiency due to a higher likelihood of co-feeding transmission for *LBS* and a shorter time span between infected and non-infected ticks feeding on the same animal.

## Methods

### Study area

The study area is along the west coast of Norway (see Additional file 1). The topography is rugged with summits reaching 1000–1500 m a.s.l. just a few kilometers from the sea. The vegetation is within the boreonemoral vegetation zone. Forests are dominated by Scots pine (*Pinus sylvestris*) on marginal soils, with alder (*Alnus incana*) dominating in richer soils, and birch (*Betula* spp.) dominating at higher elevations. There are scattered stands of Norway spruce (*Picea abies*) planted by forestry mainly on better soils at low elevation. Climate is colder with increasing altitude and distance from the coast. There are dense populations of red deer in the area. The number of harvested red deer (in 2011) was 0.7-2.4 red deer per km^2^ in the municipalities included here, which corresponds to ~3.5-12.0 red deer per km^2^. The red deer populations are partially migratory [50]. Roe deer and moose were absent from our study site. There are livestocks in several of the areas, mainly free-ranging sheep (*Ovis aries*) during summer. Other available large hosts (>1 kg) are red fox (*Vulpes vulpes*), marten (*Martes martes*), mountain hare (*Lepus timidus*) and domestic cats. Densities of these are not known, but the impression is that there are low densities of hare and marten, while red fox are more common. Information regarding densities of smaller hosts such as voles, mice and birds are unavailable.

### Tick collection

Questing *Ixodes ricinus* adult and nymphal ticks were sampled in May and August 2011 by aid of the cloth lure or “flagging” method [51] in connection with a study of variation in tick abundance in relation to red deer habitat use [50]. Sampling was based on known information on home ranges of red deer, with each transect (sampling unit) representing typically one red deer home range, but in some cases overlapping several home ranges. The spatial distribution of transects is given in Additional file 1. We used a towel (50*100 cm) attached to the end of a rod and dragged the towel over the vegetation allowing the questing ticks to attach. Each transect consisted of 12 survey plots with 20–50 m in-between, and each plot covered an approximately 10 m long and a 2 m wide belt; i.e. 20 m^2^. For every 2nd m after two drags on each side of the towel, ticks were counted and removed from the towel. Nymphs, adult females and adult males from each transect were pooled and killed using ethanol then dried and stored with silica beads at −20°C.

### DNA extraction and pathogen detection

We analysed prevalence of *A*. *phagocytophilum* and LBS for individual ticks using real-time PCR. First, DNA extractions were performed according to Allender et al. [52]. The protocol was optimized for ticks by modifying the incubation step of the Qiagen DN easy® 96 Blood & Tissue kit; where ticks, 2 mm zirconium oxide beads, 40 μl of proteinase K solution and 4 μl Antifoam-A (Sigma) were incubated at 56°C overnight followed by 5 minutes of bead homogenization at 30 cycles per second using a Qiagen Tissue Lyser II. Some ticks were not broken and were crushed using wooden toothpicks. The remaining 160 μl of proteinase K solution was added and the homogenized mixture was incubated for 1 hour at 56°C. Centrifugation was carried out before removing seals at any step in the protocol to prevent cross-contaminating samples. The mixture was then transferred to the DN easy plates using the method described by Allender et al. [52].

Detection of *A*. *phagocytophilum* and LBS was carried out using a multiplex real-time PCR assay developed by Courtney et al. [53] on a Roche Light Cycler® 480 Real-Time PCR instrument. Roche Light Cycler® 480 Probes Master reaction mixtures was used for real-time PCR in 10 μl reactions following the cycling conditions that were described [53]. Positive controls of *A*. *phagocytophilum* and LBS DNA were used in addition to negative controls from the extraction. The results from the instrument were then scored into one of five categories: *A*. *phagocytophilum* positive, LBS positive, positive for both, unknown or negative.

### Statistical analyses

The response variable was prevalence of either *A*. *Phagocytophilum* or of LBS, and we therefore used logistic regression. Our potential covariates were stage of tick (adult female, adult male or nymph), age of tick (adult vs. nymph), (log) abundance of tick in transect in May, months (May vs. August), and the spatial covariates (log) distance to main coast, (log) distance to fiord and elevation. We used two measures of red deer density. (1) The number of harvested red deer (2011) at the scale of municipalities divided by the counting area of red deer habitat, cfr. [54]. This is a coarse measure of overall population density of red deer. (2) As a more local scale measure of red deer density within a 200 m buffer from the mid-point of each transect, the habitat suitability for red deer was estimated as the ratio of use relative to availability of a given habitat, which is estimated based on a large sample of GPS-marked red deer from the same areas, cfr [55]. We considered two different random terms in our model to account for potential pseudoreplication using library “lme4”. The random term “transect” accounts for the same transect being recorded in May and August, while the random term “municipality” accounts for spatial autocorrelation among transects. We initially tested in ANOVA what random term to use. Then, we used a model selection approach aiming to find the most parsimonious model based on the Akaike Information Criterion (AIC). All analyses were performed in R v. 2.15.0.

## Results

**Figure 1:**
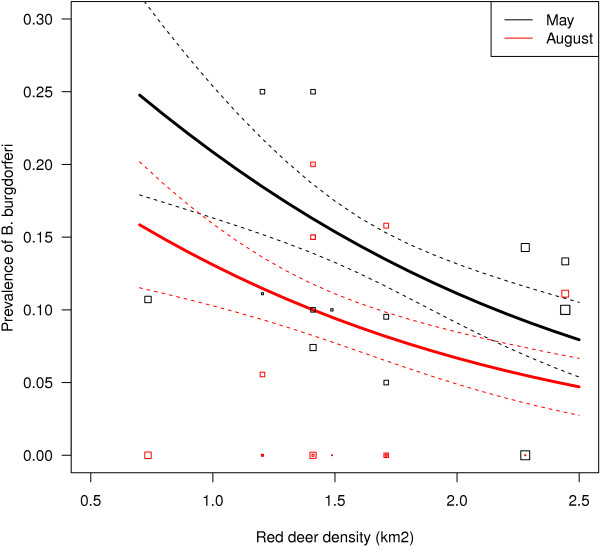
**Prevalence of *B. burgdorferi *in nymph ticks as a function of the (harvest) density of red deer at the scale of municipality in Sogn og Fjordane, Norway.** Average values for slope and distance to coast were used. Data points are raw proportions not adjusted for slope or distance to coast, while lines are predicated values (from GLM).

The overall prevalence of LBS was 10.9% in nymph (n = 686), 11.5% in adult male (n = 61) and 21.6% in adult female (n = 51) ticks (Table 1). The model including “municipality” as a random effect was a better model than the one with “transect” (p < 0.001). A model with both “transect” and “municipality” did not improve the model, as the estimated variance of the “transect” random effects was ~0. The most parsimonious model of LBS prevalence included stage of tick, month, distance to the coast, slope and density of red deer (see Additional file 1). There was higher prevalence in adult female ticks compared to adult male ticks and nymphs (Table 2). Prevalence in both stages was higher in May than in August and declined with increasing population density of red deer (Figure 1). Prevalence further decreased with increasing slope, but only tended to increase with distance from the coast (Table 2). Other covariates such as abundance of ticks, elevation and distance to fiord did not enter the best model (see Additional file 1).

**Table 1:** **An overview of the prevalence** (**n infected**) **of *A***. ***phagocytophilum *and Lyme Borreliosis Spirochate (LBS) positive adult male (M) and female (F) and nymphal ticks in May and August 2011 in Sogn & Fjordane, Norway**

**Month**	**Stage/sex**	***A. phagocytophilum***	**LBS**	**Both**	**n**
May	Nymph	0.12 (50)	0.12 (51)	0.01 (6)	423
	Adult M	0.07 (2)	0.10 (3)		29
	Adult F	0.08 (2)	0.21 (5)		24
August	Nymph	0.05 (12)	0.09 (24)	0.004 (1)	263
	Adult M	0.03 (1)	0.13 (4)		32
	Adult F	0.11 (3)	0.22 (6)		27
		0.09 (70)	0.12 (93)	0.009 (7)	798

Note that ticks with co-infection (both) are counted twice.

**Table 2:** Estimated prevalence of Lyme Borreliosis Spirochates (LBS) in ticks collected in Sogn & Fjordane, Norway from a logistic mixed effects model. adF = adult female, adM = adult male

	**Estimate**	**SE**	**Z**	**p**
Intercept	-3.2605	2.8009	-1.164	0.244
Stage (adM vs. adF)	-0.9534	0.5420	-1.759	0.079
Stage (nymph vs. adF)	-1.0043	0.3754	-2.675	0.007
Month (May vs. Aug.)	0.5729	0.2546	2.250	0.024
log(distance from the coast)	0.3959	0.2349	1.685	0.092
Slope	-0.0630	0.0174	-3.626	0.000
Red deer density	-0.8010	0.4088	-1.959	0.050

Random term was “municipality”.

The overall prevalence of *A*. *phagocytophilum* was 9.0% in nymphs (n = 686), 4.9% in adult male ticks (n = 61) and 9.8% in adult female ticks (n = 51). These differences were not significant, as stage of ticks did not enter the best model (see Additional file 1). The best random effect was “transect”, being a clearly better model than with “municipality” (p < 0.001). We found no evidence of a strong spatial structure in prevalence of *A*. *phagocytophilum*, as the most parsimonious model included only month as a predictor (see Additional file 1). There was markedly higher prevalence of *A*. *phagocytophilum* in May (11.3%) compared to August (5.0%) (Z = 2.799, p = 0.005).

We recorded 0.87% ticks with co-infection of LBS and *A*. *phagocytophilum* in all nymphs (Table 1). The overall correlation among transects in prevalence of LBS and *A*. *phagocytophilum* was moderate (r = 0.41).

## Discussion

Ticks and tick-borne diseases have increased in Europe in recent decades with severe implications for human and animal health, understanding factors determining tick abundance and prevalence of pathogens is, therefore, of major interest. Our study reveals two main patterns; that prevalence of LBS declines as red deer density increases, while there is no apparent relationship between prevalence of *A*. *phagocytophilum* and red deer density. The first finding is consistent with predictions from the host competence hypothesis concerning LBS, also termed the dilution effect [39]. However, the host competence hypothesis predicts increases in prevalence of *A*. *phagocytophilum* with increasing deer density, but this prediction was not supported. Earlier studies in Europe found no [25,27] or even positive relationship between roe deer [26] or red deer [56] density and prevalence of LBS. A problem for inference from these studies, as well as our own study, is that the ratio of Competent: incompetent hosts have not been determined explicitly. Variation in spatial density of deer does not automatically imply a change in ratio of competent: incompetent hosts, if the same factors driving deer abundance also drive variation in rodent abundance. However, in our case as in Scotland [56], much of the variation in red deer density is driven by harvesting management, thus likely reflecting a change in ratios of rodent: deer hosts rather than mainly habitat productivity differences.

Overall prevalence of *A*. *phagocytophilum* varies considerably across Europe. Our estimates (8.8%) are above the reported 1.6% for Italy/Slovenia [57], 1.9% in Luxembourg [58], 1.3% in Switzerland [59], and 3.2% in Hanover, Germany [60]. Estimates are close to the 8.7% reported in Leipzig, Germany [61], but lower than the 20.5% reported in Spain [62]. Our estimate of prevalence of *A*. *phagocytophilum* was higher than the 4.5% reported in an earlier study pooling various areas distributed over much of south Norway [63,64]. However, they reported the highest values in Hitra at the west coast (up to 19.4%), with known high abundance of red deer [64]. Low prevalence in ticks may nevertheless cause high infection rates. In Belgium, the prevalence was only 3% in questing ticks, but a 21.7% in feeding ticks on cattle was reported [65]. The prevalence of LBS (10.9% in nymphs) found in our study was within the range typically reported in other studies from Europe [58]. Higher prevalence in adult females on the one hand, and lower prevalence in adult males and nymphs on the other as we found for LBS, was reported in Austria [66] and Switzerland [67], for *A*. *phagocytophilum* in Luxembourg [58] and for both pathogens in France [68]. This was attributed to an interaction between the ticks and the bacteria, with female ticks taking a larger blood meal [68], rather than to host composition. Our mean prevalence of LBS (12%) was somewhat lower than reported along the south coast of Norway (21.4%) [34], but within the wide range of prevalence (0-25%) from a study with scattered populations across southern Norway [31] and elsewhere in Europe [69]. Though with a limited sample size, only *B*. *afzelii*, some *B*. *burgdorferi* sensu stricto and no *B*. *garinii* was reported in the coastal zone of Norway, pointing to a rodent reservoir of LBS [31].

We found no correlation between spatial variation in local questing tick abundance and prevalence of LBS or *A*. *phagocytophilum* in ticks. Prevalence of both *A*. *phagocytophilum* and LBS was much higher in May than in August, consistent with a Swedish study [45] and a study from Luxembourg [58]. This may at first seem consistent with the temporal component of the tick abundance hypothesis, as the abundance of questing nymphal ticks declined markedly over the season [50]; see also [70], assuming that the development from one stage to another takes approximately one year, so that the questing tick population experienced a similar tick density back in time when they had their last blood meal. Also, further south in Norway, a peak in prevalence was observed later in the season when tick abundance peaked in that region [34]. However, in Scotland, nymphal abundance also decreased over the season while prevalence of LBS increased [56]. The relationship between prevalence of LBS, tick abundance and seasonality thus seems to differ among areas, and the tick abundance hypothesis linked to co-feeding seems, thus, not to be an overall predictor of prevalence.

Host composition and host reservoir competence may potentially play a role in the seasonal pattern. Throughout the questing season, the composition of the questing tick population varies according to the relative amount of tick stages that either emerge from moulting after a blood meal, some months earlier in the same season or emerge after behavioural diapause from the unfed stage or after developmental diapause from engorged and non-moulted stage [71]. The specific composition of the questing tick population and the time of their last blood meal may, therefore, influence the seasonal pattern of prevalence. During spring, densities of rodents are low, while high reproduction from spring onwards substantially increases population sizes towards fall. Also birds such as thrushes have large clutches increasing the population size from spring to autumn, and migratory stress can reactivate latent *Borrelia* infection and hence increase the reservoir competence of these species [72]. In contrast, red deer populations in Norway may increase by only some 15% from spring to fall due to females producing a single offspring each year. The ratio of rodent: deer and thrush: deer hosts, therefore, is likely much higher during fall. Given a certain mortality rate of ticks through the summer, a larger proportion of the ticks questing in May have likely had their last meal during the previous late summer or early fall compared to ticks questing in August. The same seasonal decline was, however, found for prevalence of *A*. *phagocytophilum* in *Ixodes ricinus* ticks, though there are indications of rodent *A*. *phagocytophilum* ecotypes not being transmitted by this tick species [46] while deer is considered as being a competent reservoir host for at least some subpopulations [37]. That we found co-infection of both pathogens in nymphs might suggest rodents or birds also play a role for transmission of *A*. *phagocytophilum* as indicated by a North American study [73], or that individual larvae have fed on multiple hosts.

A number of other processes may potentially explain seasonal variation in prevalence, such as effects of infection on tick behavior and survival [74,75] and possibly the survival of the pathogen within the ticks over the season [76]. Firstly, ticks with infestation of LBS have larger fat stores and are more active than uninfected ticks [74,75,77]. It has also been suggested that infected ticks have a higher probability to undergo quiescence in order to avoid dry periods [58]. It may be speculated that the prevalence of infected ticks increases after environmental bottlenecks due to higher survival [77,78]. In the study area, having moist summers, but a relatively harsh winter, the prevalence of both pathogens may increase over the winter. Secondly, it may be speculated that the survival of the bacteriae within the ticks is negatively affected when moulting and activity occurs in the warmest period of the year when the immune system of the tick is likely to be more effective at killing pathogens. If so, prevalence would also be lower in fall. Lastly, differences between areas may then arise if newly emerged ticks in some areas (e.g. Scotland) are more likely to start questing in fall, while they in other areas (e.g. Norway) go into diapause if they have more energy stores.

## Conclusion

In conclusion, our study provides further support to a dilution effect of high deer density on LBS in a northern ecosystem in Europe, similar to what has been reported elsewhere [9,18-20]. Clearly, the overall disease risk might nevertheless increase even with reduced prevalence; due to deer possibly increasing tick abundance [13,79]. There was no clear association between deer density and prevalence of *A*. *phagocytophilum*. We cannot yet fully explain the seasonal decline in prevalence of both LBS and *A*. *phagocytophilum* from May to August, though several theories seem plausible. This may require further studies of the transmission cycle of the pathogens, host and tick dynamics, in particular the survival rates of the pathogens within the ticks and the influence of infection on tick questing behavior and life history.

## Competing interests

The authors affirm that they have no competing interests.

## Authors’ contributions

AM, HV and BY designed the study. LQ performed field work. WRE performed laboratory work. AM and HV carried out data analysis. AM drafted the manuscript. All authors read and approved the final version of the manuscript.

## Supplementary Material

Additional file 1: Table A1 Results from model selection of the prevalence of *B*. *burgdorferi* in Sogn & Fjordane, Norway. 1 = variable included in model. AIC = Akaike Information Criterion. ΔAIC = difference in AIC relative to the best model. “Municipality” was fitted as a random term. Click here for file
