# Supplementary material for: Spatial and seasonal variation in the prevalence of Anaplasma phagocytophilum and Borrelia burgdorferi sensu lato in questing Ixodes ricinus ticks in Norway
Source: Parasit Vectors. 2013 Jun 20;6:187. doi: 10.1186/1756-3305-6-187 (PMC3691722; doi:10.1186/1756-3305-6-187)
Supplement: Additional file 1: Table A1 — Results from model selection of the prevalence of B. burgdorferi in Sogn & Fjordane, Norway. 1 = variable included in model. AIC = Akaike Information Criterion. ΔAIC = difference in AIC relative to the best model. “Municipality” was fitted as a random term. [file 1756-3305-6-187-S1.docx]

Additional file 1

**Table A1 Results from model selection of the prevalence of *B. burgdorferi* in Sogn og Fjordane, Norway**. 1 = variable included in model. AIC= Akaike Information Criterion. ΔAIC = difference in AIC relative to the best model. “Municipality” was fitted as a random term.

| Stage of tick | log(abundance of ticks+1) | month | altitude | log(distance from the coast) | log(distance from the fiord) | slope | Habitat suitability for red deer | Density of red deer | AIC | ΔAIC |
| --- | --- | --- | --- | --- | --- | --- | --- | --- | --- | --- |
| 1 | 1 | 1 | 1 | 1 | 1 | 1 | 1 | 1 | 573.2 | 4.2 |
| 1 | 1 | 1 | 1 | 1 | 1 | 1 | 1 |  | 574.2 | 5.2 |
| 1 | 1 | 1 | 1 | 1 | 1 | 1 |  | 1 | 574.1 | 5.1 |
| 1 | 1 | 1 | 1 | 1 | 1 |  |  | 1 | 582.4 | 13.4 |
| 1 | 1 | 1 | 1 | 1 |  | 1 |  | 1 | 572.1 | 3.1 |
| 1 | 1 | 1 | 1 |  |  | 1 |  | 1 | 572.6 | 3.6 |
| 1 | 1 | 1 |  | 1 |  | 1 |  | 1 | 570.5 | 1.5 |
| 1 | 1 |  |  | 1 |  | 1 |  | 1 | 573.4 | 4.4 |
| 1 |  | 1 |  | 1 |  | 1 |  | 1 | 569.0 | 0 |
|  |  | 1 |  | 1 |  | 1 |  | 1 | 572.5 | 3.5 |

**Table A2. Results from model selection of the prevalence of *A. phagocytophilum* in Sogn og Fjordane, Norway**. 1 = variable included in model. AIC = Akaike Information Criterion. ΔAIC = difference in AIC relative to the best model. “Transect” was fitted as a random term.

| Stage of tick | log(abundance of ticks+1) | month | altitude | log(distance from the coast) | log(distance from the fiord) | slope | Habitat suitability for red deer | Density of red deer | AIC | ΔAIC |
| --- | --- | --- | --- | --- | --- | --- | --- | --- | --- | --- |
| 1 | 1 | 1 | 1 | 1 | 1 | 1 | 1 | 1 | 483.5 | 14.6 |
| 1 | 1 | 1 | 1 | 1 | 1 | 1 | 1 |  | 481.9 | 13.0 |
| 1 | 1 | 1 | 1 | 1 | 1 | 1 |  |  | 480.0 | 11.1 |
| 1 | 1 | 1 | 1 | 1 | 1 |  |  |  | 478.8 | 9.9 |
| 1 | 1 | 1 | 1 | 1 |  |  |  |  | 477.0 | 8.1 |
| 1 | 1 | 1 | 1 |  |  |  |  |  | 475.5 | 6.6 |
| 1 | 1 | 1 |  |  |  |  |  |  | 473.5 | 4.6 |
| 1 | 1 |  |  |  |  |  |  |  | 479.1 | 10.2 |
| 1 |  | 1 |  |  |  |  |  |  | 471.7 | 2.8 |
|  |  | 1 |  |  |  |  |  |  | 468.9 | 0.0 |

**Figure A1** **A map of the study area in Sogn og Fjordane, Norway with prevalence of**

**(A) *A. phagocytophilum* and (B) *B. burgdorferi* at the scale of transects.**


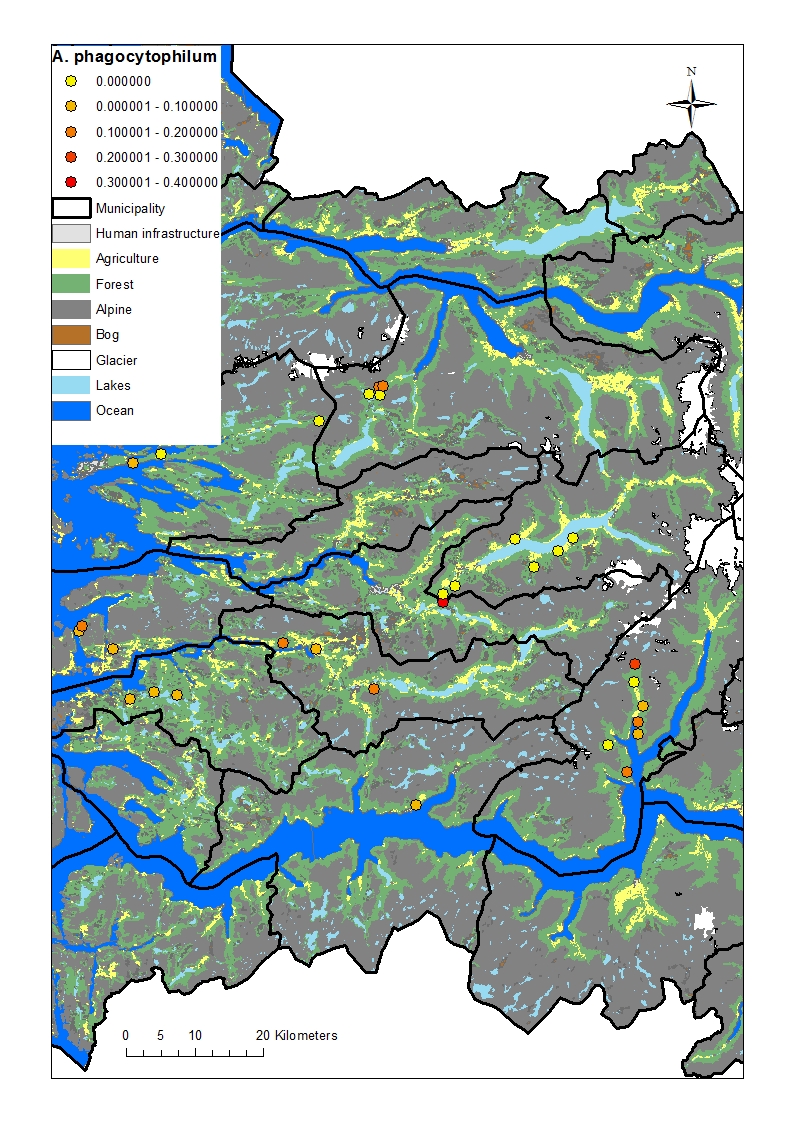


Fig. 1A


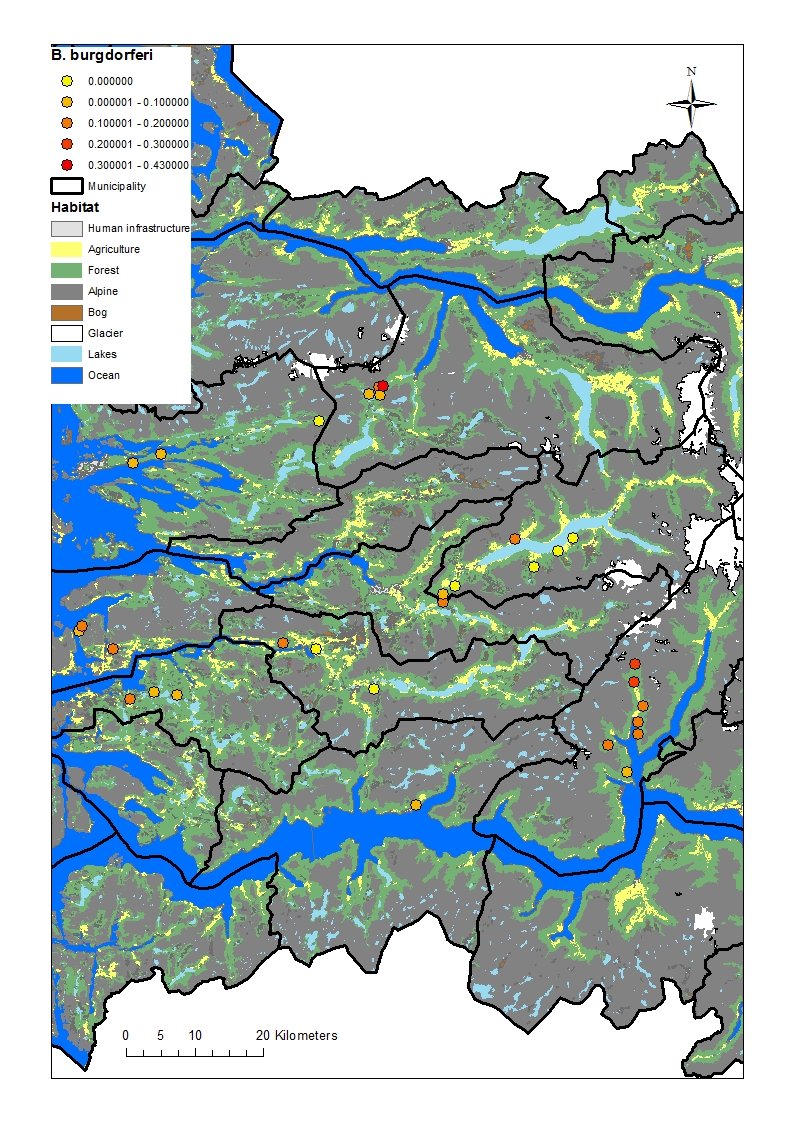


Fig. 1B
